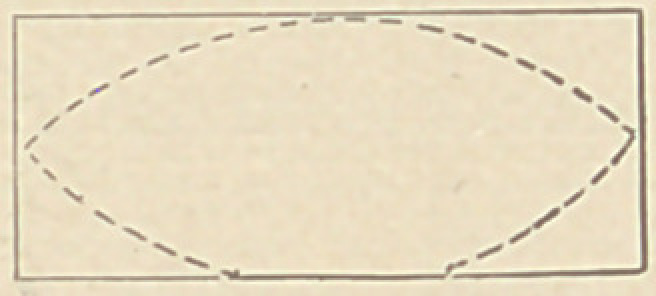# Current News and Opinion

**Published:** 1888-03

**Authors:** 


### Current News and Opinion.

### CORRESPONDENCE.

The following is presented as a model for querists. It is comprehensive, definite, and what is of more importance, it is brief. As it was evidently suggested by the report of the December meeting of the Central Dental Association of Northern New Jersey, it was submitted to the essayist of that meeting, and his most pronounced critic, for equally terse answers, which follow it. Of course, circumstances largely govern the course to be pursued. A case at present under our care will illustrate this. It is that of a child of eleven, the symmetry of whose unusually fair face is marred by unequal development of the maxillæ. The lower, which is too prominent for the upper, contains sound first molars. The upper corresponding teeth are very badly decayed, yet their extraction would intensify the partial disfigurement, and hence we think good practice demands that they should be retained at almost any hazard. Were the decayed teeth in the lower jaw, they would be unhesitatingly extracted.

EDITOR.

*Editor Independent Practitioner:*

SIR:—We have had quite a number of articles in the journals recently on "Extraction of the First Molar." Suppose the patient is eleven or twelve years old, the second molar has *not* erupted, the first molar has a dead pulp, or, perhaps, is abscessed, and is sound except one cavity leading into the pulp chamber. Would you treat and fill, or extract it and trust to the coming forward of the second molar to take the place of the first? Is it advisable to have a pulpless tooth in so young a patient? By answering the above in your next issue you will oblige,

J. H. PARSONS, D. D. S.

BOULDER, Colorado, Feb. 8, 1888.

### ANSWER OF DR. WELD.

In the first place, a sixth year molar, such as is described, pre-supposes a condition of things which would seem to call for extraction; such a tooth abscessed is not generally sound with the exception of "one cavity." The principle which involves the preservation of any tooth is sometimes lost sight of in exceptional cases, where judgment is necessary, and one cannot very well decide without first seeing the patient. I know of no good reason why a pulpless tooth, properly treated, should not be allowed to remain in the mouth of so young a patient.

G. W. WELD.

### ANSWER OF DR. TENISON.

I cannot answer Dr. Parson's questions intelligently without knowing the conditions of the other teeth, but will say it is not advisable to have a pulpless tooth, especially when it is abscessed, in so young a person's jaw, if it can be avoided. The extraction must rest with his judgment. When I find it necessary to extract one sixth year molar I extract all four, provided both jaws are normal.

W. D. TENISON.

*Editor Independent Practitioner :*

In your January number, Prof. Pierce makes objection to the remarks which I made at a meeting of American dentists in Coblenz, and asks for names, dates, etc. Prof. Pierce will find by consulting the same number of your journal that a slight mistake was made in the report, and that my remarks were not such as call for names. I have no intention of publishing names of graduates, from any college, who ought not to be in possession of the D. D. S., and that for reasons which must be apparent to every one.

If, however, Prof. Pierce wishes it, I will be glad to substantiate my remarks if you will lend the columns of your journal to this purpose.

In answer to the other questions of Prof. Pierce, I respond that by the "Pennsylvania School" I mean the Pennsylvania College of Dental Surgery, and I consider that a college gives a diploma improperly, or to an unworthy person, when it grants the same to anyone whose general or special education is below that which we have a right to demand, and which is universally expected from a person who holds the title of doctor in a specialty of medicine or surgery.

W. D. Miller.

Berlin, Feb. 2, 1888.

---

### A NEW AND SIMPLE MATRIX.

Dr. Samuel B. Freeman, of Chicago, sends us samples of a very simple matrix to be used in filling teeth, which he makes as they are wanted for each particular case. The material is "Taggert's" or "Tagger's" tin, which is the ordinary sheet tin, but of about 36 gauge, very flexible and soft. It is used by the manufacturers of tin cans for sealing after the tin is filled, as it is readily cut open with a knife. A piece cut to the proper width is folded down at one end with flat-nosed plyers so as to make it double, when it presents about the appearance of the outline of this diagram. With shears it is now cut, following the dotted lines, which it will be seen do not run entirely around the folded edge, enough being left to form a hinge for the two parts. It is placed in position, a wedge driven between the two leaves and the points bent and burnished against and around the teeth. As the tin presents a highly polished surface, it so reflects the light as to illuminate the cavity.

Of course the size, and to some extent the shape, will be dictated by the size of the tooth and cavity. It is a matrix that can be readily used in filling front teeth, and as it practically costs nothing and is made in a moment, it need not be used a second time It is certainly a very ingenious and effective appliance.

---

### A NEW ANÆSTHETIC.

Prof. Dr. Lewin, of Berlin, has made some experiments with a new alkaloid, Erythroflein, with which the inhabitants of the western coast of Africa poison their arrows. Two Cgms. (about one-sixth of a grain) were sufficient to destroy a dog. Rabbits required less. A two per cent. solution of this alkaloid, when applied to the eye of a cat, after fifteen to twenty minutes produced complete insensibility, lasting from one to two and a half days, without injury to the eye. Fifteen minutes after a hypodermic injection of this alkaloid into the back of a guinea-pig, Dr. L. was able to cut through the muscles of the back without the animal exhibiting any signs of pain. Under the influence of large doses the animals died in convulsions, but smaller doses will produce any degree of anæsthesia required. Probably the profession may find this substance of great value in obtunding sensitive dentine —*Zahnarztliches Wochenblatt, Jan.* 21.

---

### CHICAGO DENTAL CLUB.

The annual meeting of the Chicago Dental Club took place at the Tremont House, Jan. 23, 1888. The report of the Secretary showed an active membership of thirty-eight, an increase within the year of thirteen, with applications pending which in the near future will increase the membership to fifty. The dues of this club are but one dollar per annum, and it is desired to make it a practical working society, free from unprofitable or useless consumption of time. Two clinics have been given, at which many valuable operations and appliances were exhibited. The following officers were elected for the ensuing year:

*President*—Arthur B. Freeman

*Vice-President*—J. Austin Dunn.

*Secretary*—C Stoddard Smith.

*Treasurer*—E. M. S. Fernandez.

*Member Business Committee*—W. G. Stowell.

The regular meetings are held on the fourth Monday in each month, at the Tremont House. C. Stoddard Smith, Secretary.

---

### CENTRAL DENTAL ASSOCIATION OF NORTHERN NEW JERSEY.

The regular annual meeting was held on Monday evening, Feb 20th. The following were elected officers for the ensuing year:

*President*—Geo. E. Adams, South Orange.

*Vice-President*—Oscar Adelberg, Elizabeth.

*Secretary*—J. Allen Osmun, Newark.

*Treasurer*—Chas. A. Meeker, Newark.

*Executive Committee*—S. C. G. Watkins, Montclair; B. F. Luckey, Paterson; C. S. Stockton, Newark; C. F Holbrook, Newark; W. P. Richards, Orange.

---

### ILLINOIS STATE DENTAL SOCIETY.

The twenty-fourth annual meeting of the Illinois State Dental Society will be held at Cairo, beginning Tuesday, May 8th, and continuing four days. This point has been selected because convenient for the dentists of southern Illinois, with the hope that many who have not hitherto met with us will do so this year; also for the further reason that it is of easy access to those living in other States, south, east and west, to all of whom a cordial invitation is extended. An excellent programme has been arranged, with clinics as a special feature.

C. B. Rohland, President.

Garrett Newkirk, Secretary.

Mark Twain says many wise as well as humorous things. Here is one: "She was one of those people who are infatuated with patent medicines and all new-fangled methods of producing health or mending it. She was an inveterate experimenter in these things. When something fresh in this line came out she was in a fever, right away, to try it; not on herself, for she was never ailing, but on anybody else that came handy. She was a subscriber to all the 'Health' periodicals and phrenological frauds, and the solemn ignorance they were inflated with was breath to her nostrils. All the 'rot' they contained about ventilation, and how to go to bed, and how to get up, and what to eat, and what to drink, and how much exercise to take, and what frame of mind to keep one's self in, and what sort of clothing to wear, was all gospel to her, and she never observed that her health journals of the current month customarily upset everything they had recommended the month before. She was as simple-hearted and honest as the day was long, and so she was an easy victim. She gathered together her quack periodicals and her quack medicines, and thus armed with death, went about on her pale-horse, metaphorically speaking, with 'hell following after.' But she never suspected that she was not an angel of healing and the balm of Gilead in disguise to the suffering neighbors."

---

A medical summary correspondent says: "Nine persons out of every ten with a cinder or any foreign substance in the eye, will instantly rub the eye with one hand while hunting for the hankerchief with the other. They may, and sometimes do, remove the offending cinder, but more frequently they rub until the eye becomes inflamed, bind a handkerchief about the head and go to bed. This is all wrong. The better way is not to rub the eye with the cinder in it at all, but rub the other eye as vigorously as you like"

Certainly the rubbing of an eye with a cinder in it will do nothing more than to rub it in, while to rub the well eye may, through the well-known sympathetic action, cause such a muscular agitation and flow of the secretions as will spontaneously remove the cinder.

---

A Medical World correspondent says that the following lotion will preserve the skin from the effects of cold, prevent chaps and render the hands soft, white and smooth. It is to be used on the hands every night before going to bed, and in cold weather is to be applied before going out into the open air, the hands first being washed and dried:

℞

| | |
|---|---|
| Ol. Rosæ, | gtt xv. |
| Glycerinæ, | ℥ i. |
| Sp. Myrciæ, | fl℥ iij. |
| Ol. Cajaputi, | gtt xx. |

---

Chloroform and ether are both antagonistic to cocaine, and the inhalation of either will allay the convulsions due to a poisonous dose of the latter. On the other hand, cocaine may be used as an antidote in cases of poisoning by narcotic agents, especially such as cause great depression of the respiratory and cardiac centres.

Dr. Frank B. Darby, of Elmira, sends us specimens of stiffened paper points for drying pulp canals which are very useful. The first root which we had prepared for filling after their reception was tested with one of them, and to our surprise we found moisture at the extreme end, which was effectually removed by the points. Yet we had believed it quite dry, for the hot air syringe and other appliances had been, we thought, faithfully employed. A useful lesson was taught, and the possible cause of occasional pericemental irritations subsequent to root-filling revealed.

Dr. Darby's hard felt polishers with shellac centers, for the dental engine, will also be found exceedingly effectual in the polishing of fillings.

---

M. Defontaine, doctor in chief to the Creusot electric forges, in a paper read before the French Society of Surgeons describes the symptoms of a kind of sun-stroke to which the workmen are liable, from the intense light of more than 100,000 candle power from a few square centimeters of surface. The skin changes to a reddish brown sometimes, after two or three hours' work, and subsequently peels off. There are pains in the cheeks, neck and forehead, and notwithstanding the use of colored glasses, there is a flow of tears for twenty-four hours, the victims are blind to objects in common daylight for some minutes, and perfect vision is not restored for hours.

---

Dr Lenox Brown says that in extirpation of the larynx, one-third of the patients die in a few days, one-third in a few months, and none live longer than thirteen or eighteen months.—*American Lancet.*

In the number of this journal for August, 1885, we gave an account of a case of total extirpation of the larynx by Prof. Roswell Park, of the University of Buffalo. The patient, a physician of sixty, is alive and quite well to this day, two years and eight months after the operation We believe it to be true, however, that a large proportion of patients on whom this operation has been performed have died within a short time.

---

Dr. A. P. Southwick, of Buffalo, was appointed by the Governor a member of the commission to investigate and report the most humane and practical method of carrying into effect the sentence of death in capital cases. That commission has made its report to the legislature, and recommends the substitution of death by an electric shock for that by hanging. That portion which presents the practical reasons for the change was prepared by Dr. Southwick, and his argument is certainly a strong one, and well worth a careful study, whatever may be the predilections of the reader for the old-fashioned neck-stretching of hardened criminals.

---

Albany Medical Annals celebrates the new year and the commencement of its ninth volume by changing its size, its form and its management. Formerly it was published as the Organ of the Albany County Medical Society. Henceforth it will have a broader field, and be emphatically a journal of medicine. We shall look for its appearance in the future with even more of interest than in the past.

READERS OF THIS JOURNAL who have occasion to use, or to recommend to their friends or patients, an emulsion of Cod Liver Oil, should not fail to try that which is prepared by the Charles H. Phillips Chemical Co., whose advertisement appears on the second cover page of the INDEPENDENT PRACTITIONER. It is a perfect emulsion of the finest quality of Cod Liver Oil, and the latter is so completely digested that even the microscope will show only the most minute globules. It mixes with water as perfectly as milk, is pleasant to the taste, and is readily assimilated. We have tested its merits and know something of its value. F.

---

THE OHIO STATE JOURNAL OF DENTAL SCIENCE, for January, contains an excellent portrait of its editor, Dr. Geo. Watt, who is so widely known and respected in dentistry. *Items of Interest*, also, gives a counterfeit presentment of its editor in its January number. Both men are veterans, who have done good service in dentistry, each in his respective field. When the editor of this journal gets to be as good looking as his respected seniors, and has labored as long and successfully, the INDEPENDENT PRACTITIONER might have some excuse for presenting his picture.

---

THE UNEXPECTEDLY LARGE DEMAND for Prof. Stowell's Atlas of Histology in connection with subscriptions to the INDEPENDENT PRACTITIONER, entirely exhausted the first edition about the first of February, ult. A second was in process of preparation, but some unexpected delays occurred, so that it was not ready for delivery until about the fifteenth. This will account for the failure to receive the book promptly by some subscribers. It has now been forwarded to all who are entitled to it. If any have failed to receive it, they should notify our Buffalo office immediately.

---

CHICAGO has eleven medical colleges recognized by the State Board of Health. Among them is a school of dermatology. The number of professors exceeds a hundred. It is said that not a third of them receive any compensation for their work. When it is remembered that of lecturers, demonstrators and instructors there are half a hundred who receive no compensation, it will be seen that the medical professors in Chicago do not become bloated bondholders from their fees as teachers.—*American Lancet.*

---

TO SUBSCRIBE for a live dental journal costs only four-and-a-quarter cents a week, or about three-quarters of a cent a day! Compare this insignificant sum with the amount of money paid for cigars, newspapers, and street car rides, ye who think you "cannot afford" to take a dental journal! What will pay a dentist better than to keep himself supplied with the leading periodicals which relate directly to his practice?

---

IN DENTISTRY, as in every other profession, we have men who take ten times more trouble to get friends and patients than to get knowledge. They live in a fever of unrest, unless every day brings its new acquaintances, but they are content with the experience of the past, and occasional scraps picked up by the way.—*Dr. Beers.*

Franklin Leonard Pope, an authority in electrical affairs, contributes to the March number of *Scribner's Magazine* a paper on the "Electric Motor and its Applications," which is a complete account in brief compass of the origin and development of the use of electricity as a motive power. It is fully illustrated. This is an article of special value to dentists, and is well worth the subscription price of a volume

---

Seabury & Johnson supply antiseptic napkins of canton flannel that are very convenient for dental use. They are about eight inches square, but may be readily cut to smaller sizes, and are useful, not only as substitutes for the ordinary mouth napkin, but for wiping instruments, and for numberless other dental purposes. As they cost but two dollars per hundred, they are thrown away when soiled.

---

The editor of this journal will pay a liberal price in cash for the following numbers of dental journals, or he will exchange others for them with any who have files which they wish to complete.

The Dental Register, Vol. III, Nos. 1, 2, 3. Vol VI, No. 1.

The American Journal of Dental Science—third series. Vol. VII, Nos. 7, 10. Vol. VIII, No. 7.

---

The New York Odontological Society seems to be having a series of meetings this season which are both interesting and instructive. We learn that the society is reviving its old enthusiasm, and that the Executive Committee have arranged with a number of gentlemen of well-known ability to be present at future meetings and read papers that cannot fail to be productive of interest to our specialty.

---

Dr. Miller's article, as published in the last number of this journal, contained a few errors, the correction of which in the proof did not appear in the text. The names of Professors Paetsch and Sauer were mispelled, and on page 72 the *Nervus trigeminus* was metamorphosed into "Nervous Trigeminus," a change which might subject the author to the charge of bad scholarship.

---

Claudius Ash & Sons, of 30 East 14th Street, New York, have commenced the importation of the German Wolrab gold foil, and are prepared to furnish it to dentists in any quantity. No other gold has been found quite equal to this for the rotation method, while it is excellent for any use. Its virtues are, however, too well known to need comment.

---

The Waltham Emery Wheel Co. are furnishing corundum wheels and points for dentists' use that are of a superior quality. We have tested them and find that they cut readily and easily, do not heat disagreeably, and last surprisingly. As they are intended to be used dry, they present advantages for operative work.

---

"The Imperial Alloys," advertised in this journal, on account of the death of Mr. Glover, will in future be known as "The Russell Alloys" "The rose by any other name would smell as sweet," and the character of the alloys is not altered by the change of name.

---

The Kansas State Dental Society meets at Topeka, the last Tuesday in April, 1888. Arrangements are in progress for a more than usually interesting meeting.

## Figures and Tables

**Figure f1:**